# Pseudomonas aeruginosa Citrate Synthase GltA Influences Antibiotic Tolerance and the Type III Secretion System through the Stringent Response

**DOI:** 10.1128/spectrum.03239-22

**Published:** 2023-01-05

**Authors:** Hao Chen, Xuetao Gong, Zheng Fan, Yushan Xia, Yongxin Jin, Fang Bai, Zhihui Cheng, Xiaolei Pan, Weihui Wu

**Affiliations:** a State Key Laboratory of Medicinal Chemical Biology, Key Laboratory of Molecular Microbiology and Technology of the Ministry of Education, Department of Microbiology, College of Life Sciences, Nankai University, Tianjin, China; Emory University School of Medicine

**Keywords:** *Pseudomonas aeruginosa*, citrate synthase, stringent response, type III secretion system

## Abstract

Carbohydrate metabolism plays essential roles in energy generation and providing carbon skeletons for amino acid syntheses. In addition, carbohydrate metabolism has been shown to influence bacterial susceptibility to antibiotics and virulence. In this study, we demonstrate that citrate synthase *gltA* mutation can increase the expression of the type III secretion system (T3SS) genes and antibiotic tolerance in Pseudomonas aeruginosa. The stringent response is activated in the *gltA* mutant, and deletion of the (p)ppGpp synthetase gene *relA* restores the antibiotic tolerance and expression of the T3SS genes to wild-type level. We further demonstrate that the intracellular level of cAMP is increased by the stringent response in the *gltA* mutant, which increases the expression of the T3SS master regulator gene *exsA*. Overall, our results reveal an essential role of GltA in metabolism, antibiotic tolerance, and virulence, as well as a novel regulatory mechanism of the stringent response-mediated regulation of the T3SS in P. aeruginosa.

**IMPORTANCE** Rising antimicrobial resistance imposes a severe threat to human health. It is urgent to develop novel antimicrobial strategies by understanding bacterial regulation of virulence and antimicrobial resistance determinants. The stringent response plays an essential role in virulence and antibiotic tolerance. Pseudomonas aeruginosa is an opportunistic pathogen that causes acute and chronic infections in humans. The bacterium produces an arsenal of virulence factors and is highly resistant to a variety of antibiotics. In this study, we provide evidence that citrate synthase GltA plays a critical role in P. aeruginosa metabolism and influences the antibiotic tolerance and virulence. We further reveal a role of the stringent response in the regulation of the antibiotic tolerance and virulence. The significance of this work is in elucidation of novel regulatory pathways that control both antibiotic tolerance and virulence in P. aeruginosa.

## INTRODUCTION

Bacterial pathogenesis is largely dependent on virulence factors, whose functions include adherence to host cells/tissues, killing or interfering with functions of host cells, nutrient acquisition, etc. (1, 2). Meanwhile, bacteria possess a variety of intrinsic and acquired antibiotic resistance mechanisms. In addition, a small population of bacteria form dormant persister cells that can survive lethal doses of bactericidal antibiotics (3), which contributes to recalcitrant and chronic infections (4, 5) and provides a reservoir for the development of antibiotic-resistant mutants (6).

Pseudomonas aeruginosa is a Gram-negative bacterial pathogen that causes various acute and chronic infections in humans (7). The type III secretion system (T3SS) of P. aeruginosa plays an important role in acute infections (8). It directly injects effector proteins into host cells, leading to cell death or malfunction (9). Expression of the T3SS genes is regulated by the master regulator ExsA; the *exsA* gene is localized in the operon composed of *exsC*, *exsE*, *exsB*, and *exsA*. Transcription of *exsA* is driven by its own promoter (P*_exsA_*) and the upstream *exsC* promoter (P*_exsC_*), which are regulated by Vfr/cAMP and ExsA, respectively (10, 11).

In most bacteria, antibiotic tolerance and persister cell formation are regulated by the alarmone molecule guanosine tetra- and pentaphosphate (p)ppGpp (12–14), whose homeostasis is regulated by RelA and SpoT (15). RelA is a monofunctional (p)ppGpp synthetase that is activated by amino acid starvation (16). RelA binds to uncharged tRNA, and then the complex enters the A site of the ribosome, which activates the (p)ppGpp synthetase activity of RelA (17, 18). SpoT is a bifunctional enzyme with both (p)ppGpp hydrolase and synthetase activities (19). Studies in Escherichia coli demonstrated that the activity of SpoT can be regulated by acyl carrier protein (ACP) (20). SpoT binding to acyl-ACP activates the (p)ppGpp hydrolysis activity, whereas binding to uncharged ACP promotes (p)ppGpp synthetase activity (20). Besides controlling persister formation, the stringent response has been demonstrated to influence P. aeruginosa virulence (21).

Recent studies demonstrated that carbon sources influence bacterial tolerance to antibiotics (22). Intermediates of the tricarboxylic acid (TCA) cycle, such as fumarate and succinate, sensitize P. aeruginosa to tobramycin (23). By screening P. aeruginosa strains defective in carbon metabolism, we previously demonstrated that triosephosphate isomerase *tpiA* mutations reduce the cytotoxicity and resistance to aminoglycoside antibiotics (24). Here in this study, we demonstrate that mutation in the citrate synthase gene *gltA* increases the cytotoxicity and antibiotic tolerance by activating the stringent response. In addition, our results reveal a role of the stringent response in regulating the intracellular cAMP level in P. aeruginosa.

## RESULTS

### Mutation of *gltA* increases the expression of the T3SS genes and bacterial survival under antibiotic treatment.

**FIG 1:**
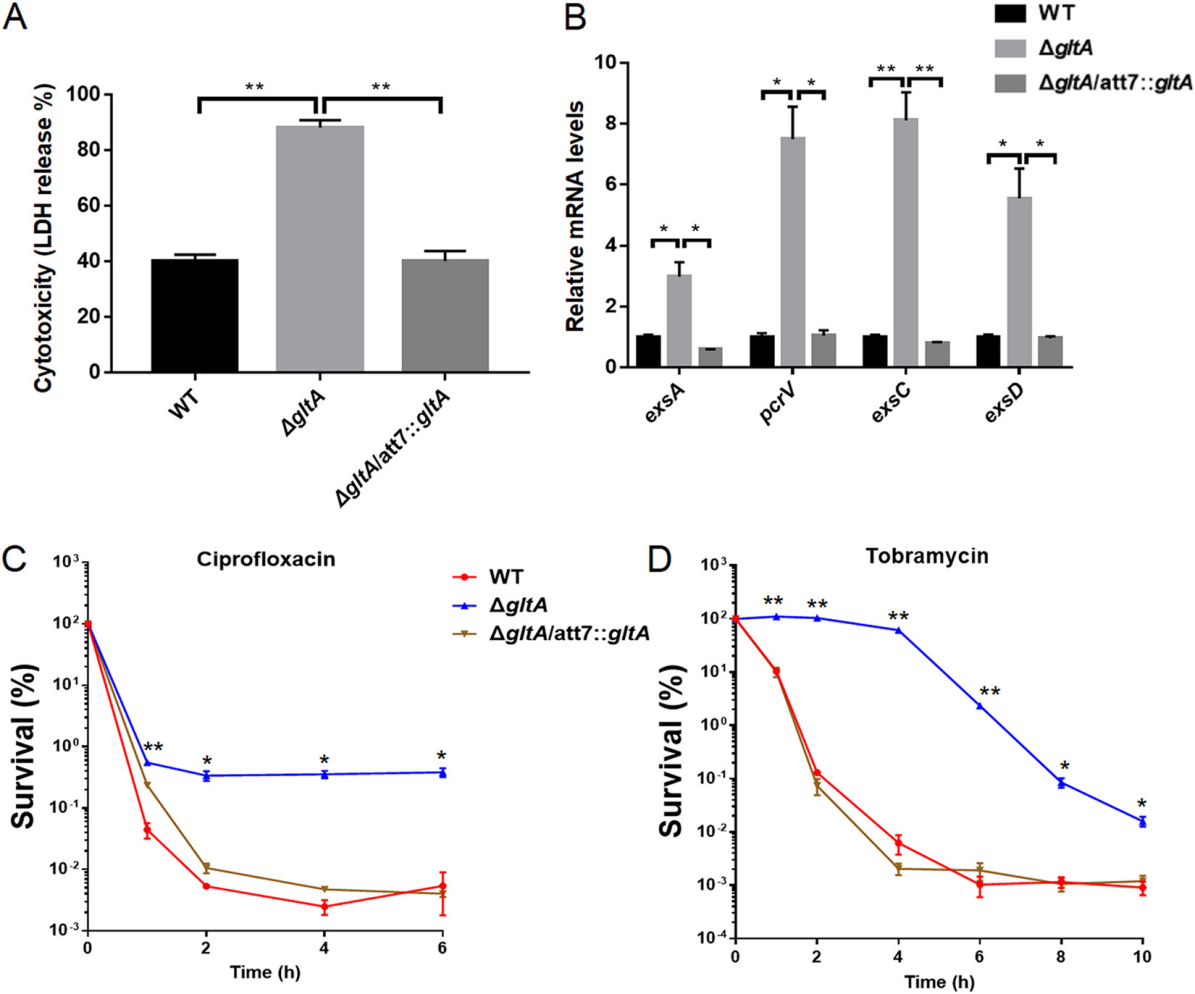
Roles of GltA in cytotoxicity and antibiotic tolerance. (A) Cytotoxicity of wild-type (WT) PA14, the Δ*gltA* mutant, and the complemented strain. The relative cytotoxicity was determined by the lactate dehydrogenase (LDH) release assay. (B) The relative mRNA levels of the T3SS genes were determined by real-time PCR. *, *P* < 0.05; **, *P* < 0.01, by Student’s *t* test. (C and D) Bacterial survival after treatment with 0.5 μg/mL ciprofloxacin (C) or 4 μg/mL tobramycin (D) in LB. Data represent the mean ± standard deviation from assays performed in triplicate and are representative of three independent experiments with similar results. *, *P* < 0.05; **, *P* < 0.01, compared to wild-type PA14 or the complemented strain at each of the corresponding time points by one-way ANOVA.

We previously found that a transposon insertion in the *gltA* gene enhanced bacterial cytotoxicity against A549 cells (24). To verify the role of *gltA* in bacterial cytotoxicity, we deleted the *gltA* gene in wild-type PA14, which increased cytotoxicity. Complementation with a *gltA* gene reduced cytotoxicity to wild-type levels (Fig. 1A). In P. aeruginosa, the cell contact cytotoxicity is mainly contributed by the T3SS. Reverse transcription-quantitative PCR (qRT-PCR) results demonstrated that mutation in the *gltA* gene enhanced expression of the T3SS genes, including the regulatory genes *exsA*, *exsC*, and *exsD* and the structural gene *pcrV* (Fig. 1B).

Next, we examined the role of GltA in PA14 resistance and tolerance to various antibiotics, including ciprofloxacin, tobramycin, azithromycin, meropenem, and polymyxin B. The *gltA* deletion did not affect the resistance (MICs) (see Table S1 in the supplemental material). We then examined the bacterial survival in antibiotic killing assays. Treatment with ciprofloxacin resulted in typical biphasic killing curves. The survival of the Δ*gltA* mutant was higher than those of the wild-type strain and the complemented strain at the slow killing phase (Fig. 1C), indicating a higher percentage of persister cells. Treatment of the wild-type PA14 and the complemented strain with tobramycin resulted in rapid killing within the initial 2 h, followed by slower curves. However, a slow killing curve was observed on the Δ*gltA* mutant in the initial 4 h, followed by a rapid killing curve. After 10 h, survival of the Δ*gltA* mutant was approximately 20-fold higher than those of the wild-type PA14 and the complemented strain (Fig. 1D). These results suggest a role of GltA in the tolerance and persistence under tobramycin treatment.

### Mutation of *gltA* decreases the bacterial membrane permeability.

**FIG 2:**
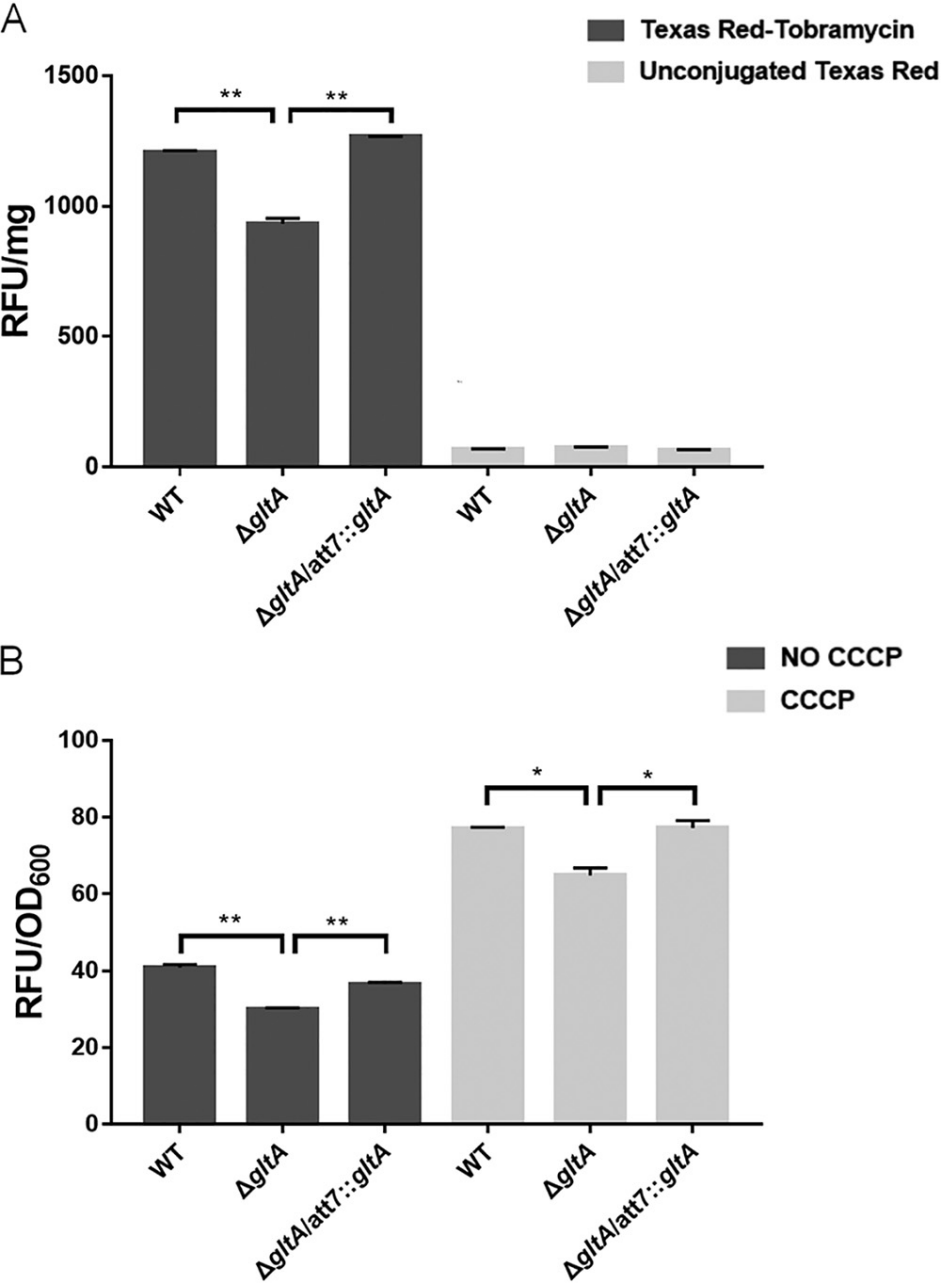
GltA affects membrane permeability. (A) Uptake of tobramycin-Texas Red (TbTR) or Texas Red (TR) by wild-type PA14, the Δ*gltA* mutant, and the complemented strain. Relative fluorescence units (RFU) were normalized by the total protein amounts. Data represent the mean ± standard deviation from assays performed in triplicate and are representative of three independent experiments with similar results. **, *P* < 0.01, by Student’s *t* test. (B) Uptake of EtBr by the indicated strains in the presence or absence of 100 μM CCCP. Data represent the mean ± standard deviation of assays performed in triplicate and are representative of three independent experiments with similar results. *, *P* < 0.05; **, *P* < 0.01, by Student’s *t* test.

To understand the mechanism that increases tobramycin tolerance in the Δ*gltA* mutant, we measured the intracellular accumulation of a Texas Red-labeled tobramycin (designated TbTR). Compared to wild-type PA14, there was an approximately 1.5-fold-smaller amount of TbTR in the Δ*gltA* mutant, whereas there was no difference in the intracellular accumulation of unconjugated Texas Red (Fig. 2A). We then utilized an ethidium bromide (EtBr) internalization assay to examine membrane permeability (25). A smaller amount of EtBr was internalized by the Δ*gltA* mutant in the absence or presence of the efflux pump inhibitor carbonyl cyanide *m*-chlorophenyl hydrazone (CCCP) (Fig. 2B). In combination, these results suggest that reduction in membrane permeability might play a role in the increased survival of the Δ*gltA* mutant.

### Mutation of *gltA* activates the stringent response.

**FIG 3:**
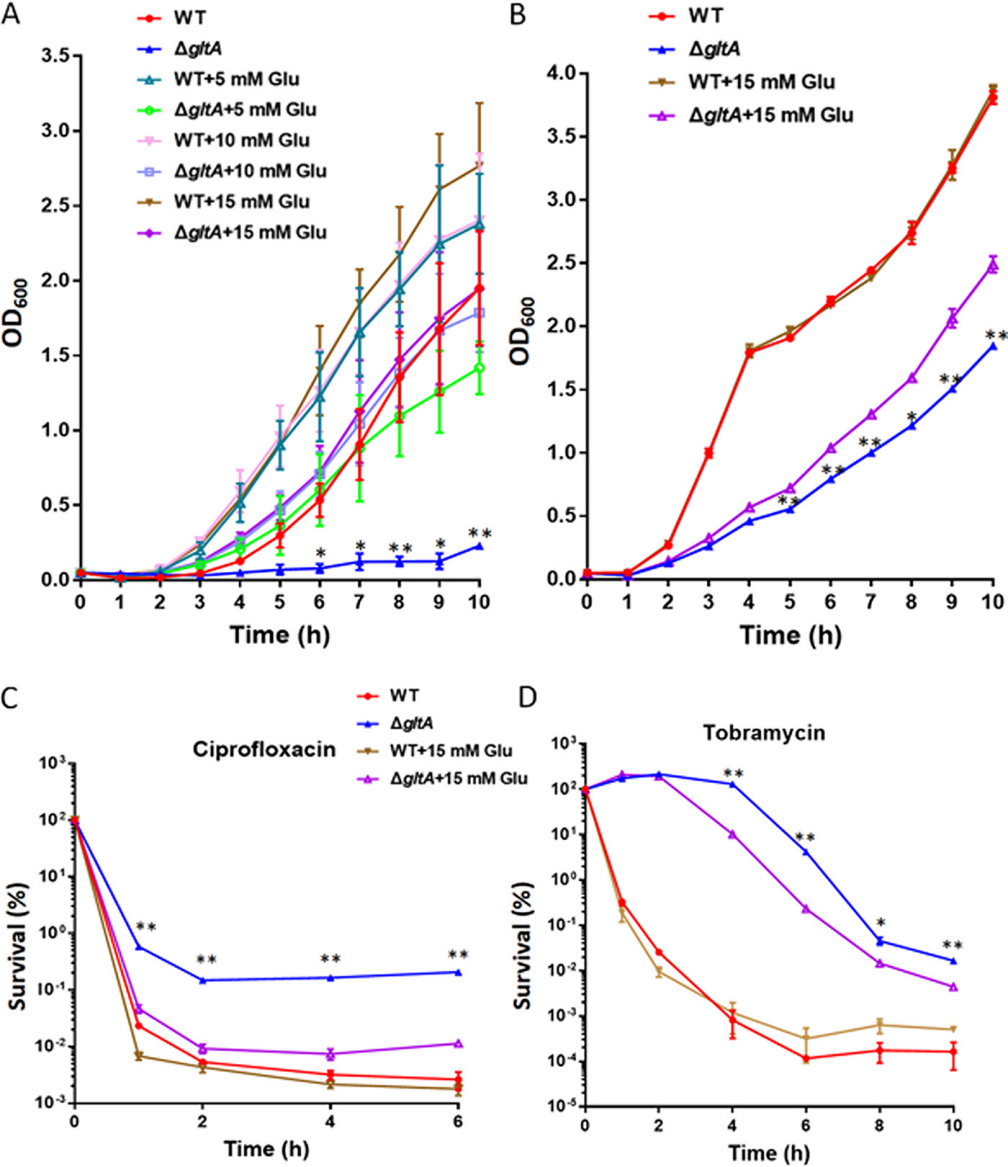
Supplementation of glutamate restores growth and antibiotic susceptibility of the Δ*gltA* mutant. (A) Bacterial growth in M9 minimal medium with increasing concentrations of glutamate was monitored by measuring OD_600_ every hour for 10 h. *, *P* < 0.05; **, *P* < 0.01, compared to wild-type PA14 or the Δ*gltA* mutant supplemented with 5, 10, or 15 mM glutamate at the corresponding time points by one-way ANOVA. (B) Bacterial growth in LB medium with or without 15 mM glutamate was monitored by measuring OD_600_ every hour for 10 h. *, *P* < 0.05; **, *P* < 0.01, compared to the Δ*gltA* mutant supplemented with 15 mM glutamate at each of the corresponding time points by one-way ANOVA. (C and D) Bacterial survival after treatment with 0.5 μg/mL ciprofloxacin (C) or 4 μg/mL tobramycin (D) in LB with or without 15 mM glutamate. Data represent the mean ± standard deviation from assays performed in triplicate and are representative of three independent experiments with similar results. *, *P* < 0.05; **, *P* < 0.01, compared to the Δ*gltA* mutant supplemented with 15 mM glutamate at each of the corresponding time points by one-way ANOVA. Glu, glutamate.

*gltA* encodes the citrate synthase that catalyzes the condensation of oxaloacetate and acetyl coenzyme A (acetyl-CoA) to form citrate, which is the first reaction of the TCA cycle. Mutation of *gltA* resulted in a growth defect when glucose was the sole carbon source (Fig. S1). However, growth of the Δ*gltA* mutant was partially restored when citric acid was the sole carbon source (Fig. S1). Besides the critical role in energy metabolism, the TCA cycle provides precursors of certain amino acids. A previous study in Klebsiella pneumoniae demonstrated that mutation in *gltA* leads to auxotrophy in the glutamate amino acid family (26). Meanwhile, amino acid starvation is known to activate the stringent response (27). We thus hypothesized that the increased survival of the Δ*gltA* mutant against antibiotics might be due to activation of the stringent response induced by deficiency of the glutamate family amino acids. Indeed, the Δ*gltA* mutant displayed a severe growth defect in an M9 minimal medium with glucose as the sole carbon source, which was restored by supplementation with glutamate in a concentration-dependent manner (Fig. 3A). In the LB medium, the growth rate of the Δ*gltA* mutant was lower than that of the wild-type PA14, which was partially restored by supplementation of glutamate (Fig. 3B). In addition, supplementation of glutamate reduced the survival of the Δ*gltA* mutant following treatment with ciprofloxacin or tobramycin (Fig. 3C and D).

**FIG 4:**
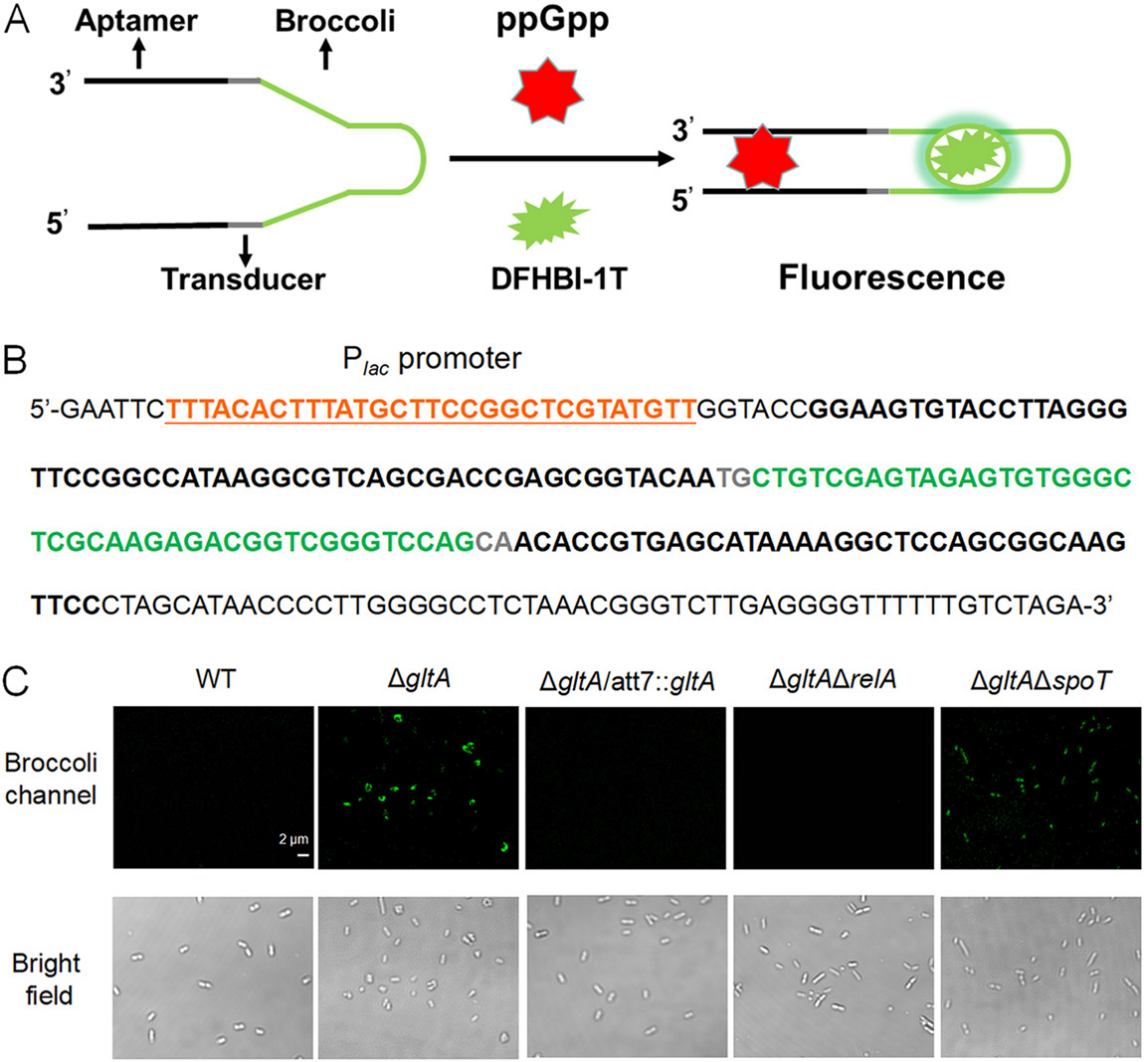
Imaging of intracellular (p)ppGpp. (A) Schematic of an RNA-based (p)ppGpp sensor. The (p)ppGpp binding aptamer, transducer, and Broccoli RNA sequences are shown in black, gray, and green, respectively. Binding between (p)ppGpp and the aptamer alters the folding of the Broccoli RNA, and when the folded aptamer binds to the dye DFHBI-1T, green fluorescence is generated. (B) The sequence of the (p)ppGpp sensor. The (p)ppGpp sensor RNA is driven by a *lac* promoter (P*_lac_*) without a CRP binding sequence (orange and underlined). (C) Confocal fluorescence imaging of cells exhibiting fluorescence from the (p)ppGpp sensor.

To examine whether the stringent response is activated in the Δ*gltA* mutant, we visualized (p)ppGpp in live bacterial cells by using an RNA-based fluorescent sensor (Fig. 4A and B) (28). Compared to wild-type PA14 and the complemented strain, the Δ*gltA* mutant exhibited stronger fluorescence (Fig. 4C). Deletion of *relA* in the Δ*gltA* mutant reduced fluorescence to wild-type levels, whereas deletion of *spoT* only slightly reduced the fluorescence of the Δ*gltA* mutant (Fig. 4C). In addition, deletion of *relA* but not *spoT* restored the growth rate of the Δ*gltA* mutant (Fig. S2). These results suggest that RelA plays a major role in the increase of (p)ppGpp in the Δ*gltA* mutant.

**FIG 5:**
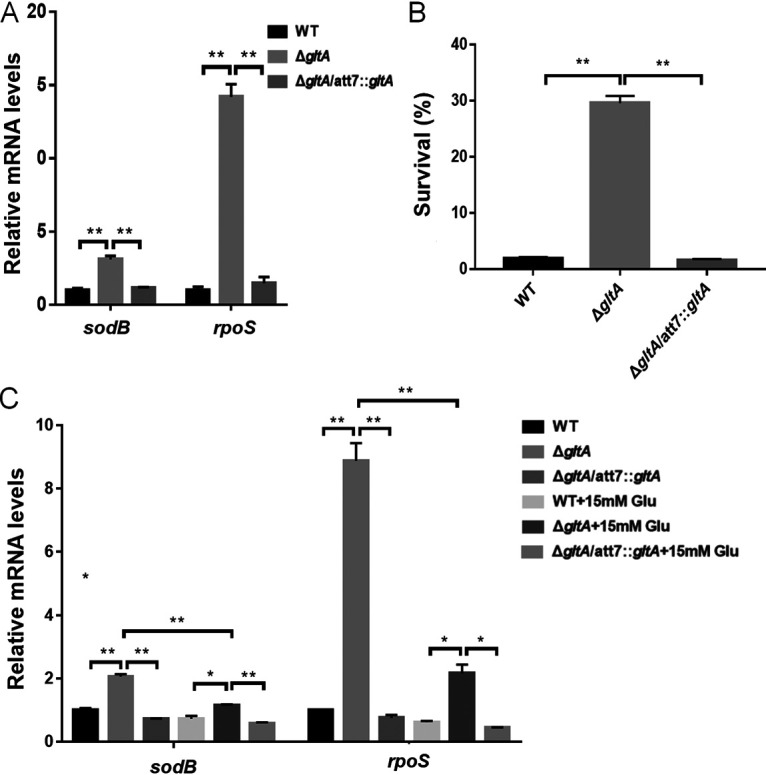
GltA affects the expression of *sodB* and *rpoS*. (A) The mRNA levels of *sodB* and *rpoS* in wild-type PA14, the Δ*gltA* mutant, and the complemented strain were determined by real-time PCR. Data represent the mean ± standard deviation from assays performed in triplicate and are representative of three independent experiments with similar results. **, *P* < 0.01, by Student’s *t* test. (B) Bacterial survival after treatment with 100 mM H_2_O_2_ in PBS for 30 min. Data represent the mean ± standard deviation from assays performed in triplicate and are representative of three independent experiments with similar results. **, *P* < 0.01, by Student’s *t* test. (C) The mRNA levels of *sodB* and *rpoS* in bacteria grown in LB with or without 15 mM glutamate were determined by real-time PCR. Data represent the mean ± standard deviation from assays performed in triplicate and are representative of three independent experiments with similar results. *, *P*  <  0.05; **, *P* < 0.01; ns, not significant by Student’s *t* test.

**FIG 6:**
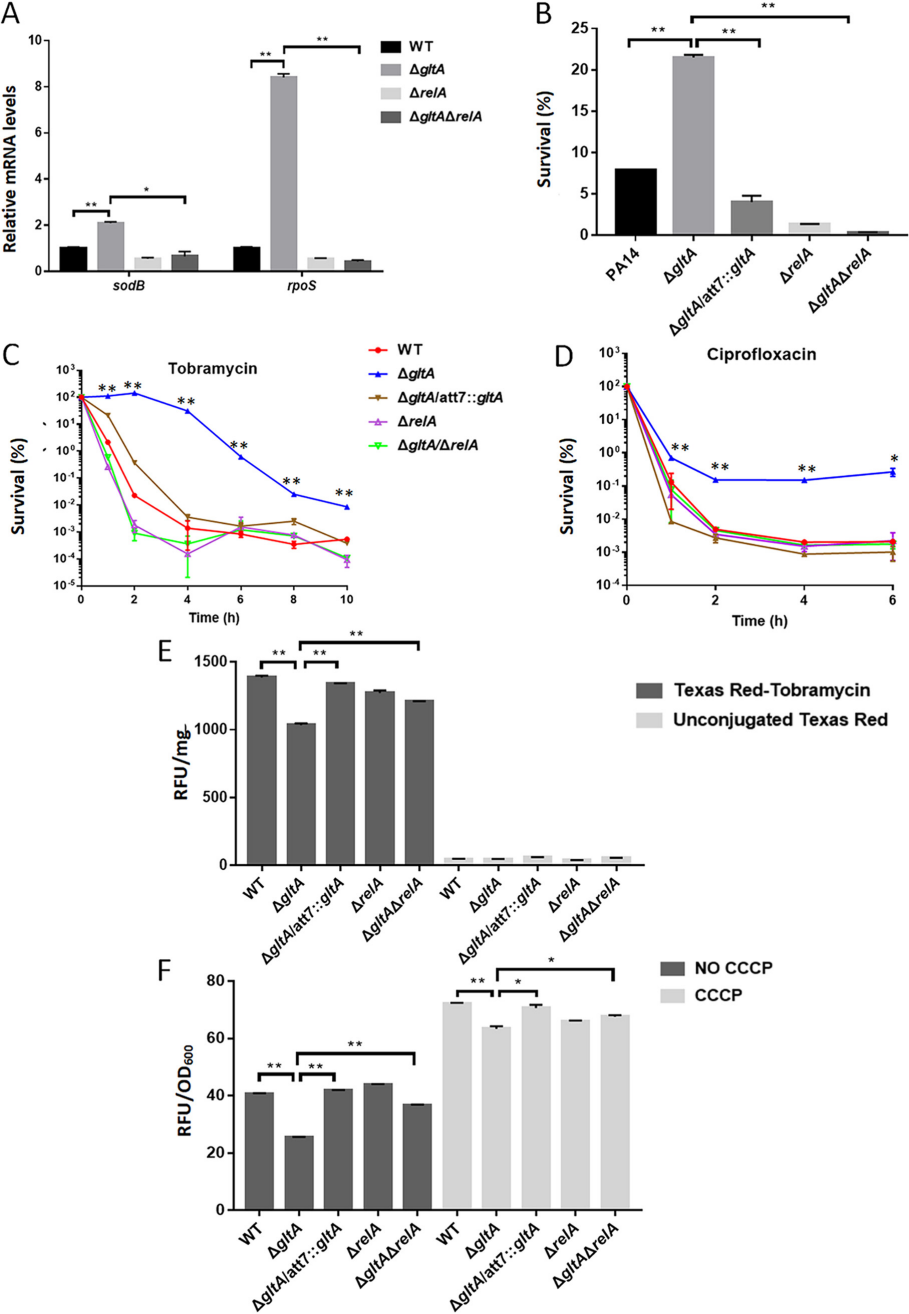
Roles of RelA in the stringent response of the Δ*gltA* mutant. (A) The mRNA levels of *sodB* and *rpoS* in the indicated strains were determined by real-time PCR. Data represent the mean ± standard deviation from assays performed in triplicate and are representative of three independent experiments with similar results. *, *P* < 0.05; **, *P* < 0.01, by Student’s *t* test. (B) Bacterial survival after treatment with 100 mM H_2_O_2_ in PBS for 30 min. Data represent the mean ± standard deviation from assays performed in triplicate and are representative of three independent experiments with similar results. **, *P* < 0.01, by Student’s *t* test. (C and D) Bacterial survival after treatment with 4 μg/mL tobramycin (C) or 0.5 μg/mL ciprofloxacin (D). Data represent the mean ± standard deviation from assays performed in triplicate and are representative of three independent experiments with similar results. *, *P* < 0.05; **, *P* < 0.01, compared to the other strains at the corresponding time points by one-way ANOVA. (E) Uptake of tobramycin-Texas Red (TbTR) or Texas Red (TR) by the indicated strains. Data represent the mean ± standard deviation from assays performed in triplicate and are representative of three independent experiments with similar results. **, *P* < 0.01, by Student’s *t* test. (F) Bacterial uptake of EtBr in the presence or absence of 100 μM CCCP. Data represent the mean ± standard deviation from assays performed in triplicate and are representative of three independent experiments with similar results. *, *P* < 0.05; **, *P* < 0.01, by Student’s *t* test.

We further examined the expression of *rpoS* and *sodB* that had been demonstrated to be upregulated by the stringent response (29, 30). Mutation of *gltA* increased the mRNA levels of *rpoS* and *sodB* (Fig. 5A). Both the sigma factor RpoS and superoxide dismutase (SOD) SodB contribute to bacterial tolerance to oxidative stresses (30). Consistent with the upregulation of *rpoS* and *sodB*, the Δ*gltA* mutant was more tolerant to H_2_O_2_ (Fig. 5B). Supplementation of glutamate in LB reduced the expression levels of *rpoS* and *sodB* in the Δ*gltA* mutant (Fig. 5C). In addition, deletion of *relA* in the Δ*gltA* mutant reduced the expression levels of *rpoS* and *sodB* (Fig. 6A) and bacterial survival following treatment with H_2_O_2_, tobramycin, or ciprofloxacin (Fig. 6B to D) and increased the uptake of TbTR and EtBr (Fig. 6E and F). In combination, these results suggest that in the Δ*gltA* mutant the RelA-mediated stringent response increases bacterial survival following treatment with tobramycin and ciprofloxacin.

### Upregulation of the T3SS genes in the Δ*gltA* mutant is caused by the stringent response.

**FIG 7:**
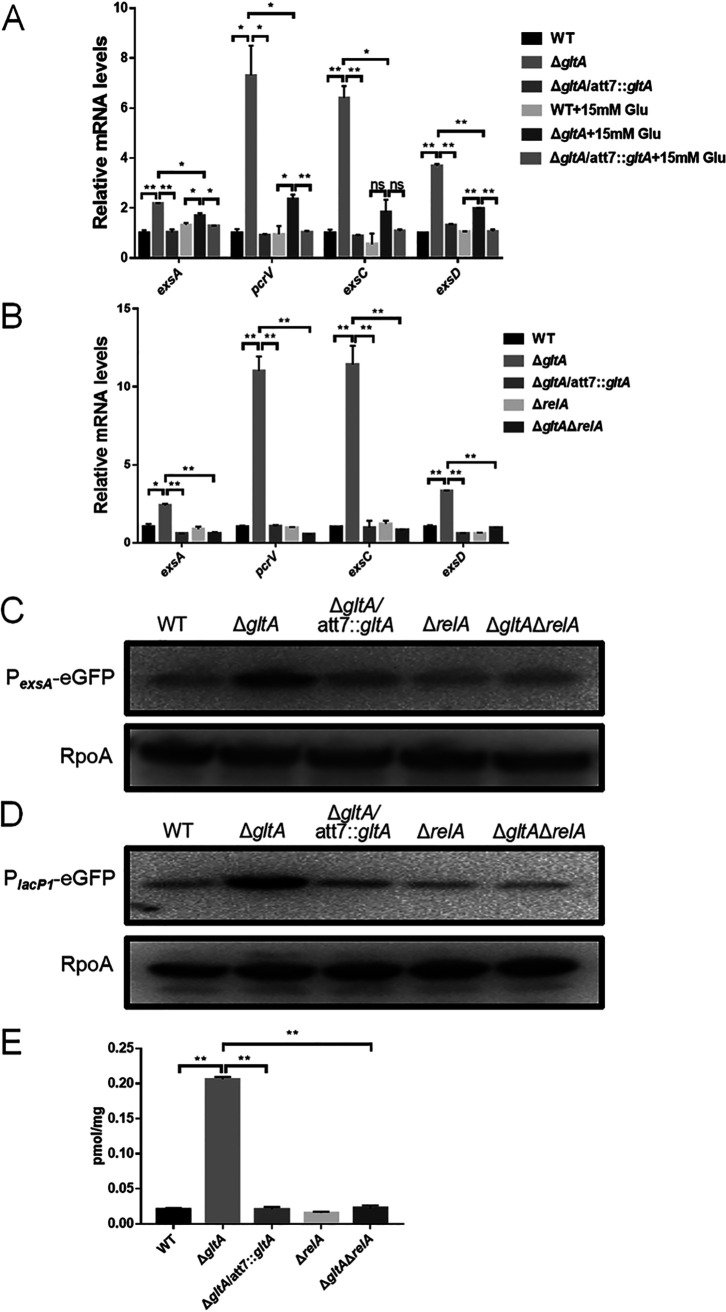
Roles of RelA in the regulation of the T3SS in the Δ*gltA* mutant. (A) The mRNA levels of the T3SS genes in bacteria grown with or without 15 mM glutamate were determined by real-time PCR. Data represent the mean ± standard deviation from assays performed in triplicate and are representative of three independent experiments with similar results. *, *P* < 0.05; **, *P* < 0.01; ns, not significant by Student’s *t* test. (B) The mRNA levels of the T3SS genes were determined by real-time PCR. Data represent the mean ± standard deviation from assays performed in triplicate and are representative of three independent experiments with similar results. *, *P* < 0.05; **, *P* < 0.01, by Student’s *t* test. (C and D) Indicated strains containing an *egfp* gene driven by the *exsA* promoter (C) or *lacP1* promoter (D) were grown in LB at 37°C. The GFP levels were determined by Western blotting with RpoA as the loading control. (E) The intracellular cAMP levels in the indicated strains. Data represent the mean ± standard deviation from assays performed in triplicate and are representative of three independent experiments with similar results. **, *P* < 0.01, by Student’s *t* test.

We next explored the mechanism that upregulates the T3SS genes in the Δ*gltA* mutant. Supplementation of glutamate reduced the mRNA levels of *exsA*, *exsC*, *exsD*, and *pcrV* in the Δ*gltA* mutant (Fig. 7A). This result led us to hypothesize that the stringent response might play a role in the upregulation of the T3SS genes. Indeed, deletion of *relA* in the Δ*gltA* mutant restored the expression of the T3SS genes (Fig. 7B).

To understand the mechanism of the upregulation of the T3SS genes, we determined the activity of the *exsA* promoter. By using a P*_exsA_*-*gfp* transcriptional fusion, we found that mutation of *gltA* increased the P*_exsA_* activity (Fig. 7C). The mRNA level of *vfr* was not affected by the *gltA* mutation (Fig. S3), indicating a possible increase of intracellular cAMP levels. The *lacP1* promoter activity had been used to indicate intracellular levels of cAMP (31). Thus, we constructed a P*_lacP1_*-*gfp* transcriptional fusion and transferred it into an adenylate cyclase-defective mutant (Δ*cyaA* Δ*cyaB*) (32). Expression of the green fluorescent protein (GFP) was lower in the Δ*cyaA* Δ*cyaB* mutant than that in wild-type strain (Fig. S4), verifying the correlation between the intracellular cAMP levels and the *lacP1* promoter activity. The GFP level was higher in the Δ*gltA* mutant than in the wild-type PA14 (Fig. 7D). Deletion of *relA* in the Δ*gltA* mutant reduced the expression of the *gfp* gene driven by P*_exsA_* and P*_lacP1_* (Fig. 7C and D). We then directly determined the intracellular cAMP levels. Deletion of *gltA* increased the intracellular cAMP level, which was restored to wild-type levels by deletion of *relA* (Fig. 7E). Meanwhile, the mRNA levels of the adenylate cyclase genes *cyaA* and *cyaB* and the cAMP phosphodiesterase gene *cpdA* (32) were not altered by the mutation of *gltA* (Fig. S5). In combination, these results suggest that the stringent response upregulates the T3SS genes by increasing the intracellular level of cAMP in the Δ*gltA* mutant.

To examine whether activation of the T3SS is specific to the GltA deficiency-induced stringent response, we used serine hydroxamate (SHX) to mimic amino acid starvation (28). SHX treatment increased the intracellular (p)ppGpp level in wild-type PA14 (Fig. S6A) but did not affect the mRNA levels of *exsA*, *exsC*, and *pcrV* (Fig. S6B), indicating that the T3SS is not activated by the SHX-induced stringent response.

## DISCUSSION

In this study, we found that deletion of the citrate synthase gene *gltA* activated the stringent response, which increased tolerance to antibiotics and H_2_O_2_ as well as expression of the T3SS genes. Mutation of *gltA* resulted in growth defects in M9 minimum medium, which were partially rescued by supplementation with glutamate or citric acid. These results suggest a glutamate deficiency in the Δ*gltA* mutant, presumably due to insufficient carbon skeletons for the synthesis of amino acids. Although glutamate supports the synthesis of glutamate family amino acids (glutamine, proline, histidine, and arginine) and citric acid fuels the TCA cycle, we suspected that a defect in the condensation between acetyl-CoA and oxaloacetate might result in aberrant levels of acetyl-CoA and oxaloacetate, thus disturbing the normal metabolism pathways and hindering bacterial growth.

Amino acid deficiency can increase the intracellular pool of uncharged tRNA, which triggers the RelA-mediated stringent response. Previous studies in P. aeruginosa demonstrated that inactivation of the stringent response (by deletion of *relA* and *spoT*) increased membrane permeability and reduced tolerance to oxidative stresses and antibiotics, which are due to a decrease in superoxide dismutase (SOD) activity (30, 33). Our results demonstrated that the *gltA* mutation increased expression of *sodB* and reduced the membrane permeability to and uptake of tobramycin, which were restored to the wild-type level by deletion of *relA*. In addition, supplementation of glutamate restored the expression level of *sodB* and membrane permeability. Previously, Erickson et al. demonstrated that mutation of *relA* abolished (p)ppGpp production under amino acid starvation (34). Previous studies demonstrated that elevated levels of ppGpp increase bacterial survival following ciprofloxacin treatment (35). In combination, these results suggest that the enhanced antibiotic tolerance of the Δ*gltA* mutant might be due to RelA-mediated activation of the stringent response triggered by amino acid starvation. It has been demonstrated that induction of reactive oxygen species (ROS) might be another bacterium-killing mechanism of antibiotics (36). Thus, upregulation of SOD by the stringent response might confer protection by increasing the ability to detoxify ROS. Martins et al. demonstrated that the stringent response increases the expression of superoxide dismutase, which decreases membrane permeability and drug internalization, thus contributing to gentamicin and ofloxacin tolerance in P. aeruginosa (30). We demonstrated that mutation of *gltA* increased the stringent response and the expression of *rpoS* and *sodB* (Fig. 5A). Deletion of *relA* in the Δ*gltA* mutant reduced the expression of *rpoS* and *sodB* and increased membrane permeability (Fig. 6A, E, and F) and bacterial susceptibility to tobramycin and ciprofloxacin (Fig. 6C and D). These results suggest that the activated stringent response might increase antibiotic tolerance by reducing membrane permeability, detoxifying oxidative stress, and promoting persister formation. However, the mechanism of stringent response-mediated regulation on *rpoS* and *sodB* remains to be elucidated.

By utilizing a *relA spoT* double mutant, previous studies demonstrated that the stringent response is required for the bacterial virulence in the infection models of Drosophila melanogaster feeding, murine acute pneumonia, murine skin infection, and rat lung agar bead (21, 37, 38). The *relA spoT* double mutant was found to be defective in the production of pyocyanin, elastase, protease, and siderophores (37). The stringent response has been shown to control the quorum sensing (QS) systems in P. aeruginosa. Simultaneous mutation of *relA* and *spoT* reduced the production of the *las* and *rhl* QS signal molecules 3-oxo-dodecanoyl-homoserine lactone (3-oxo-C_12_-HSL) and lactone (C_4_-HSL) butanoyl-homoserine, whereas it increased the production of the *pqs* signal molecule 4-hydroxyl-2-heptylquinoline (HHQ) and 3,4-dihydroxy-2-heptylquinoline (PQS) (39). A recent study by Pletzer et al. demonstrated that an LESB58 Δ*relA* Δ*spoT* mutant was defective in surfing motility (40).

A previous study demonstrated upregulation of the T3SS genes in a PAO1 Δ*relA* Δ*spoT* mutant in 2YT broth (38). However, another study demonstrated downregulation of the T3SS genes in a PAO1 Δ*relA* Δ*spoT* mutant in LB broth (21). The discrepancy might be due to the growth media, as the 2YT broth contains 1.6- and 2-fold-higher concentrations of tryptone and yeast extract, respectively, and half the concentration of NaCl compared to LB broth. Here, we found that a Δ*gltA* mutation resulted in upregulation of the T3SS genes in LB, which was caused by an increase in cAMP. We further demonstrated that mutation of *relA* in the Δ*gltA* mutant reduced the cAMP level and expression levels of the T3SS genes, indicating a role of the stringent response in modulating cAMP levels. The physiological roles of cAMP have been comprehensively studied in E. coli. Besides catabolite repression, cAMP has been shown to be involved in converting active 70S ribosomes to inactive 70S monomers or 100S dimers, which is termed ribosome hibernation and contributes to bacterial survival under environmental stresses (41, 42). Ribosome-associated inhibitor A (RaiA) binds to the 70S ribosome and maintains it in an inactive form (41). Ribosome modulation factor (RMF) and hibernation promoting factor (HPF) promote dimerization of the 70S ribosomes (41). The cAMP-cAMP receptor protein (CRP) complex has been shown to directly activate the expression of *rmf* and *raiA* in E. coli (43). A recent study in E. coli demonstrated that waking of persister cells is triggered by sensing of extracellular nutrients by the chemotaxis apparatus, which reduces intracellular cAMP levels and subsequently resuscitates ribosomes (44). However, high concentrations of cAMP have been shown to reduce persister formation in E. coli (45), which might be due to the roles of cAMP in the bacterial stress response in addition to ribosome hibernation (46). Therefore, the levels of cAMP might influence the balance in persister formation and waking.

In P. aeruginosa, catabolite repression is mainly controlled by the catabolite repression control protein (Crc), the RNA chaperone Hfq, and the small RNA CrcZ instead of cAMP (47). cAMP and the CRP Vfr play important roles in the regulation of multiple virulence factors, including T3SS, the type II secretion system, swimming and twitching motilities, quorum sensing systems, etc. (48). However, it remains largely unknown whether cAMP plays a role in persister formation and waking in P. aeruginosa. Here, we found that a Δ*gltA* mutant triggers the RelA-mediated stringent response, which increased cAMP levels and subsequently the expression of T3SS genes. The expression of the cAMP synthesis and hydrolysis genes was not affected by the *gltA* mutation (see Fig. S5 in the supplemental material). Therefore, it is possible that (p)ppGpp influences the enzymatic activities of adenylate cyclases. Further studies are needed to elucidate the relationship between (p)ppGpp and cAMP in P. aeruginosa. We also found that the SHX-induced stringent response did not increase the T3SS genes’ expression, which is consistent with a previous report (38). Considering that SHX binds to and interferes with seryl-tRNA synthetases (49, 50), while mutation of *gltA* leads to defects in both the TCA cycle and synthesis of glutamate family amino acids, the upregulation of the T3SS genes in the Δ*gltA* mutant might be related to the overall metabolism. In our previous study, we found that mutations in *tpiA*, pyruvate dehydrogenase component genes *aceE* and *aceF*, and the glycerol-3-phosphate dehydrogenase gene *gpsA* decreased cytotoxicity, whereas mutation in the aldolase gene PA2843 increased cytotoxicity (38). Further studies are needed to elucidate the interrelationship between metabolism, the stringent response, cAMP, and the T3SS.

Overall, our results demonstrated that a PA14 Δ*gltA* mutant induces the stringent response, which increases tolerance to antibiotics and the expression of T3SS genes. Therefore, interruption of metabolic pathways might increase the difficulty in eliminating bacterial pathogens. It is necessary to comprehensively evaluate the bacterial phenotypes when choosing the treatment targets.

## MATERIALS AND METHODS

### Bacterial strains and plasmids.

All bacterial strains, plasmids, and primers used in this study are listed in Table S2 in the supplemental material. Gene deletion and complementation were performed as previously described (51). Detailed methods are provided in the supplemental methods.

### Cytotoxicity assay.

Cytotoxicity was measured using a lactate dehydrogenase (LDH) cytotoxicity assay kit (Beyotime, Shanghai, China). Each strain was tested in triplicate. The human lung carcinoma A549 cell line was cultured in Roswell Park Memorial Institute (RPMI) medium with 10% (vol/vol) heat-inactivated fetal bovine serum at 37°C with 5% CO_2_. Each well of a 24-well plate was inoculated with 2 × 10^5^ A549 cells and cultured overnight. Bacteria were cultured at 37°C in LB medium to an optical density at 600 nm (OD_600_) of 1. One milliliter of the bacterial cells was collected by centrifugation at 10,000 × *g* for 1 min and washed with phosphate-buffered saline (PBS). The bacteria were resuspended in 1 mL PBS, resulting in 1 × 10^9^ CFU/mL. The A549 cells were washed once with PBS and infected with the bacteria at a multiplicity of infection (MOI) of 50 in RPMI without serum for 1 h. LDH in the medium was measured using an LDH cytotoxicity assay kit (Beyotime, Shanghai, China). The control for the total LDH release was the cells incubated with the LDH release buffer (provided by the kit). Calculation of the cytotoxicity percentage was performed following the manufacturer’s instructions.

### Real-time PCR.

Overnight bacterial cultures were diluted 1:100 into fresh LB and grown to an OD_600_ of 1 in LB. Three hundred microliters of the bacterial cells was collected by centrifugation at 10,000 × *g* for 1 min, followed by RNA isolated with a bacterial total RNA kit (Zomanbio, Beijing, China). Qualities of the RNA samples were determined by measuring OD_260_/OD_280_ and OD_260_/OD_230_. Samples with both ratios of ≥2.0 were used for following experiments. A 1.0-μg amount of RNA was used to synthesize cDNA with reverse transcriptase and random primers (TaKaRa, Dalian, China). The same amount of cDNA was mixed with the SYBR II green supermix (Bio-Rad, Beijing, China) and specific primers (Table S2). Real-time PCR was performed with the CFX Connect real-time system (Bio-Rad, USA). Since (p)ppGpp has been shown to inhibit the expression of rRNA and ribosomal protein genes (52), we used previously reported housekeeping gene PA1805 as the internal control for normalization (53). Primers were designed with a software program (Primer Premier 5; Bio-Rad, USA), and the sequences ae listed in Table S2. Melting curve analyses were performed with the CFX Connect real-time system (Bio-Rad, USA) to verify the specific amplification of target regions. Each sample was tested in triplicate.

### Bacterial growth rate.

Bacteria were grown overnight in LB. One milliliter of the bacteria was collected by centrifugation and washed once with PBS and then resuspended in 1 mL PBS. Based on the OD_600_ of each strain, the same amount of the bacteria was inoculated in M9 minimal medium or LB with increasing concentrations of glutamate to achieve an initial OD_600_ of 0.05. Bacterial growth was monitored by measuring OD_600_ with a spectrophotometer (SmartSpec Plus; Bio-Rad, USA) every hour for 10 h. When the OD_600_ was above 1.0, the bacterial culture was diluted 4-fold before absorbance measurement. Each strain was tested in triplicate.

### Antibiotic susceptibility test.

The MICs were determined using the broth microdilution method in accordance with Clinical and Laboratory Standards Institute (CLSI) recommendations (54). Bacteria were cultured at 37°C in Mueller-Hinton broth (MHB) with shaking at 200 rpm until the OD_600_ reached 1.0. Each well of a 96-well microtiter plate was filled with 100 μL Mueller-Hinton broth (MHB) with serially diluted antibiotics. Then, 5 × 10^5^ CFU of bacteria in 100 μL MHB was added into each well. Each strain was tested in triplicate. The plate was incubated at 37°C for 24 h. The MIC was recorded as the lowest concentration of antibiotic that inhibits visible growth.

### Antibiotic killing assays.

Overnight cultures of wild-type PA14 and the *gltA*-complemented strain (Δ*gltA*/att7::*gltA*) were diluted 1:100 into fresh LB, and the overnight culture of the Δ*gltA* mutant was diluted 1:60 in LB. After culture for 2 h at 37°C with shaking at 200 rpm, the three strains reached an OD_600_ of around 0.4. One milliliter of the bacteria was collected by centrifugation and resuspended in the same volume of LB, resulting in approximately 4 × 10^8^ CFU/mL. Then, the bacteria were treated with individual antibiotics at indicated concentrations at 37°C with agitation at 200 rpm. For CFU counts, the bacteria were serially diluted and 15 μL of each sample was spotted on an LB plate, resulting in a detection limit of 66 CFU/mL. The plates were incubated at 37°C for 36 h before colony counting. Each sample was tested in triplicate.

### Tobramycin-Texas Red uptake assay.

Tobramycin was conjugated to Texas Red (TbTR) as previously described (23). Bacteria were grown in LB to an OD_600_ of 0.4. Then, the bacteria were incubated with 40 mg/L TbTR or Texas Red for 30 min. The bacteria were collected and washed twice with PBS, followed by lysis through sonication. Texas Red was measured with a fluorometer (Varioskan Flash; Thermo Scientific) at excitation/emission (Ex/Em) wavelengths of 595/615 nm, respectively. The total protein concentration was quantified using a bicinchoninic acid (BCA) analysis kit (Beyotime, Shanghai, China). Each strain was tested in triplicate.

### EtBr staining.

Bacterial cells were grown in LB to an OD_600_ of 0.4. Then, the bacterial cells were adjusted to a density of 8 × 10^8^ CFU/mL in PBS and stained with 30 μM EtBr for 1 h at 37°C in the dark. The EtBr staining was performed in the presence or absence of 100 μM cyanide 3-chlorophenylhydrazone (CCCP). Relative fluorescence units (RFU) of bacterial cells were measured at Ex/Em wavelengths of 490/585 nm, respectively, by a fluorometer (Varioskan Flash; Thermo Scientific). Each sample was tested in triplicate.

### H_2_O_2_ susceptibility assay.

Bacteria were grown in LB to an OD_600_ of 0.4 and then resuspended in PBS, followed by incubation with 100 mM H_2_O_2_ (Sigma) at 37°C for 30 min. The number of live bacteria was determined by serial dilution with PBS and plating on LB agar plates. Each sample was tested in triplicate.

### Cellular imaging of (p)ppGpp.

The imaging was performed as previously described with minor modifications (28). Bacteria expressing the (p)ppGpp RNA sensor were grown in LB at 37°C until OD_600_ reached 1.0. The bacteria were washed twice with PBS, followed by incubation with 200 μM (5Z)-5-[(3,5-difluoro-4-hydroxyphenyl)methylene]-3,5-dihydro-2-methyl-3-(2,2,2-trifluoroethyl)-4H-imidazol-4-one (DFHBI-1T) (MedChemExpress, USA) in PBS at room temperature for 30 min. All fluorescence images were collected with a confocal microscope (FV1200; Olympus) equipped with FV10-ASW software.

### Western blotting.

Bacteria were grown to an OD_600_ of 1.0. The same amount of proteins from each of the strains was loaded onto a 12% sodium dodecyl sulfate-polyacrylamide (SDS-PAGE) gel. Then, the proteins were transferred onto a polyvinylidene fluoride (PVDF) membrane and probed with an anti-GFP antibody (1:2,000; Cell Signaling Technology, USA) at 4°C overnight. The PVDF membrane was washed four times with PBS containing 0.1% Tween 20 (PBST), followed by incubation with a horseradish peroxidase (HRP)-conjugated secondary antibody. After washing with PBST four times, the membrane was incubated with an enhanced chemiluminescence (ECL) reagent (Millipore). The signals were visualized by a Bio-Rad molecular imager (ChemiDoc XRS). The RNA polymerase α subunit RpoA was used as a loading control with an antibody (1:2,000) from BioLegend, USA.

### cAMP assay.

The intracellular cAMP concentrations were determined with a detection kit (Cayman Chemical, USA). Bacterial cells (4 mL) were grown in LB to an OD_600_ of 1.0 prior to chilling on ice for 10 min, and then cells were harvested by centrifugation at 4°C. The bacteria were washed twice with cold 0.9% NaCl and then resuspended in 150 μL of 0.1 M HCl and incubated on ice for 15 min with occasional vortexing. After centrifugation at 13,000 × *g* for 5 min at 4°C, the cAMP concentration in the supernatant was determined with an enzyme-linked immunosorbent assay (ELISA) kit (Cayman Chemical, USA). The total protein concentration was quantified using a bicinchoninic acid (BCA) analysis kit (Beyotime, Shanghai, China). Each sample was tested in triplicate.

### Genome sequencing.

Genomic DNA of the indicated strains was purified with a DNA purification kit (Tiangen Biotec, Beijing, China). Genome sequencing was performed by Azenta Life Sciences (Suzhou, China) with the MGISEQ2000 platform. Paired-end sequenced raw reads were filtered with the fastp (v.0.20.0) preprocessor. The clean reads were mapped to the genome of PA14 using the BWA software. The Unified Genotyper calls single-nucleotide variations and insertions-deletions (SNVs/indels) with GATK (V3.8.1) software. Annotations for calling of single-nucleotide polymorphisms (SNPs) and small insertions and deletions (indels) were performed using Freebayes, snpEff, and bcftools.

### Statistical analysis.

All experiments were performed at least 3 times. Bacterial survival and growth rate results were analyzed by one-way analysis of variance (ANOVA). qRT-PCR and fluorescence density data were analyzed by Student’s *t* test.
