# Supplementary material for: Pseudomonas aeruginosa Citrate Synthase GltA Influences Antibiotic Tolerance and the Type III Secretion System through the Stringent Response
Source: Microbiol Spectr. 2023 Jan 5;11(1):e03239-22. doi: 10.1128/spectrum.03239-22 (PMC9927146; doi:10.1128/spectrum.03239-22)
Supplement: Supplemental file 1 — Supplemental material. Download spectrum.03239-22-s0001.pdf, PDF file, 1.1 MB [file spectrum.03239-22-s0001.pdf]

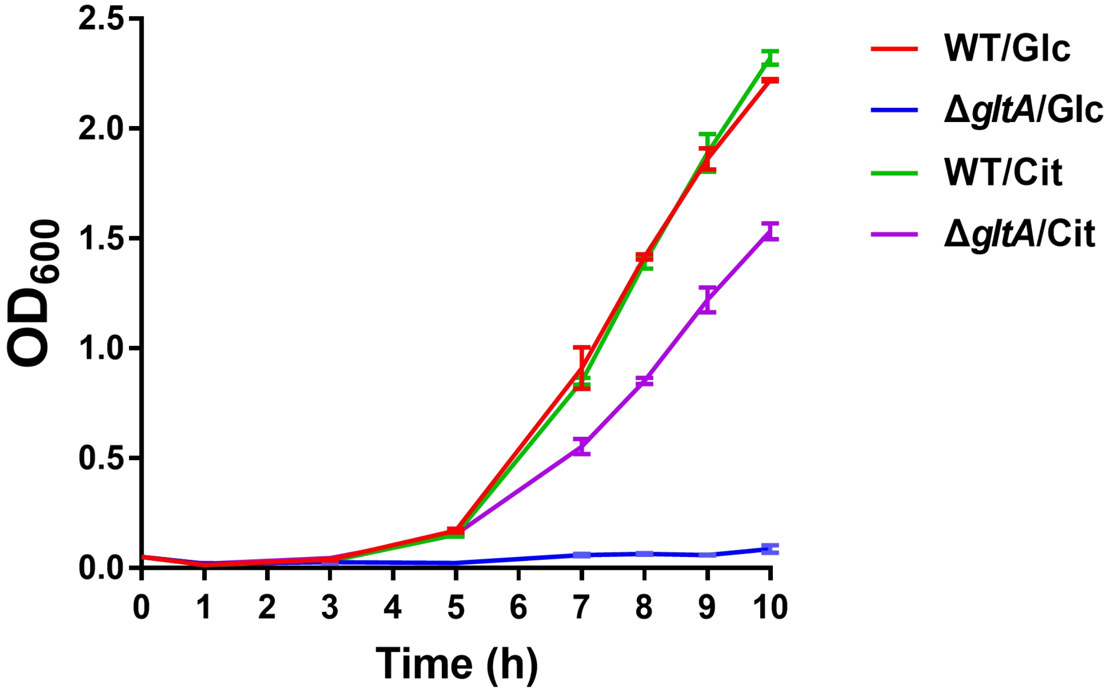

Fig. S1. Bacterial growth curves. Same amount of bacteria were inoculated in the M9 medium with glucose (Glc) or citric acid (Cit) as the sole carbon source. The bacterial growth was monitored by measuring OD_{600} every hour for 10 hours.

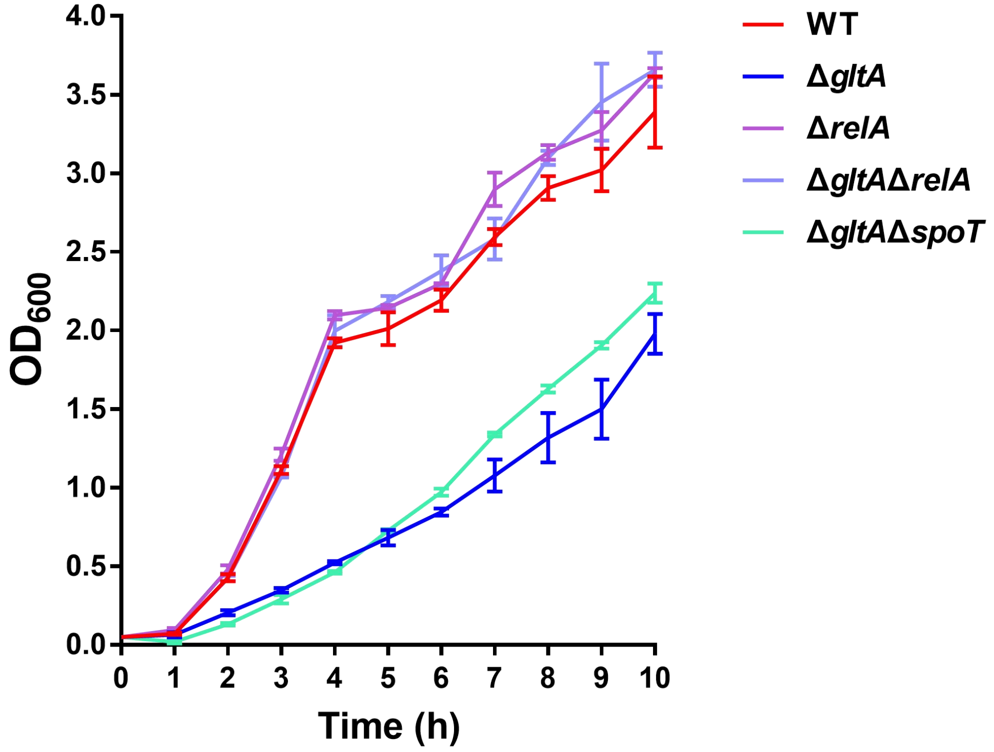

Fig. S2. Bacterial growth curves. Same amount of bacteria were inoculated in fresh LB. The bacterial growth was monitored by measuring OD_{600} every hour for 10 hours.

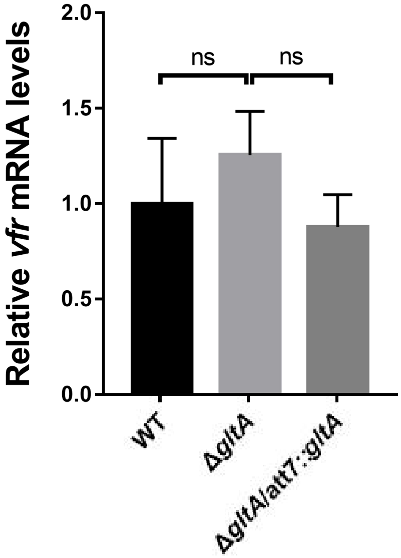

Fig. S3. Expression levels of the *vfr* gene. The mRNA levels of the *vfr* gene were determined by real-time PCR. Data represent the mean ± standard deviation performed in triplicate and are representative of three independent experiments with similar results. ns, not significant by Student's *t* test.

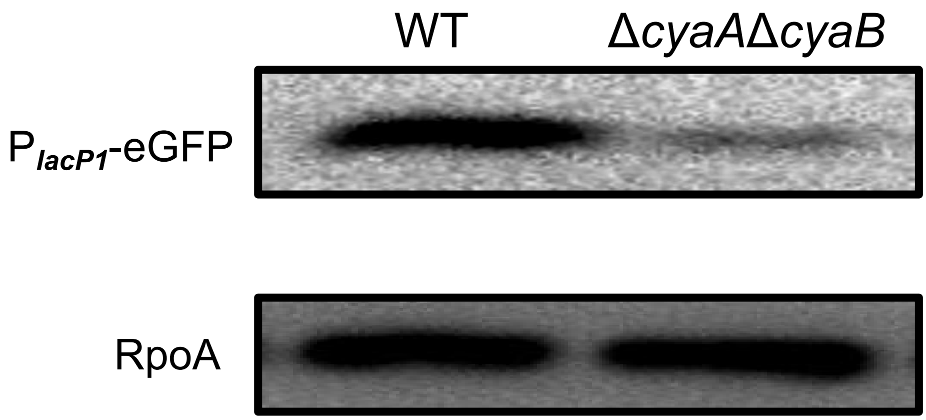

Fig. S4. Expression of the P$_{lacP1}$-*egfp*. Wild type strain and the Δ*cyaA*Δ*cyaB* mutant containing the P$_{lacP1}$-*egfp* were grown in LB at 37 °C to an OD_{600} of 1.0. The GFP levels were determined by western blot with RpoA as the loading control.

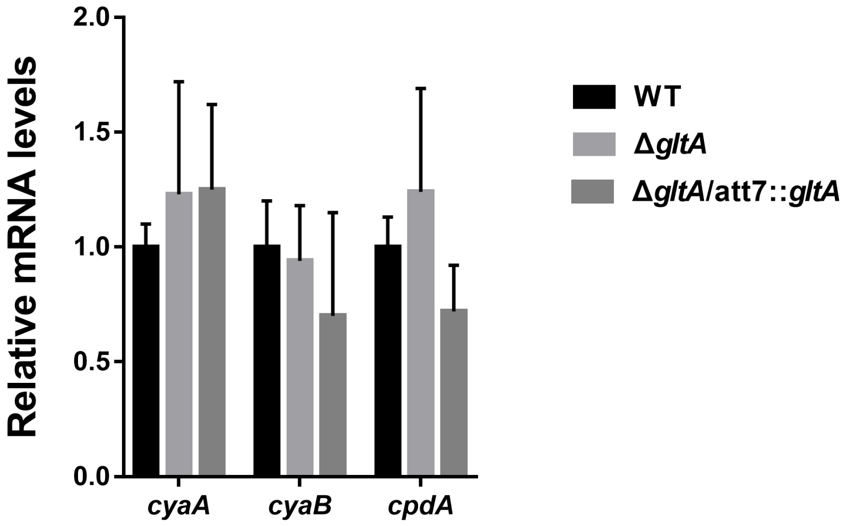

Fig. S5. Expression levels of the *cyaA*, *cyaB* and *cpdA* gene. The mRNA levels of the *cyaA*, *cyaB* and *cpdA* gene were determined by real-time PCR. Data represent the mean ± standard deviation performed in triplicate and are representative of three independent experiments with similar results. ns, not significant by Student's *t* test.

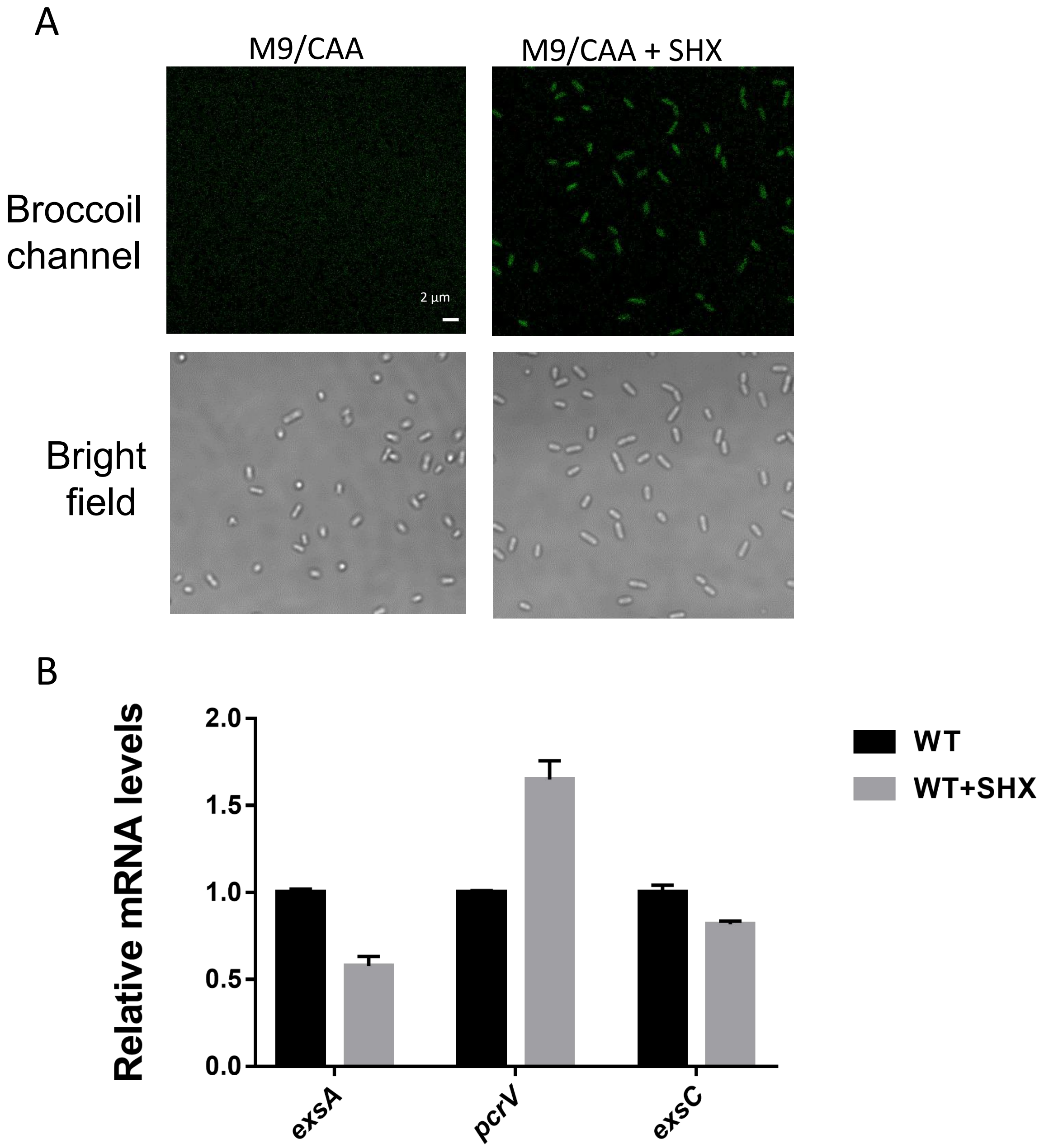

Fig. S6. **Imaging of (p)ppGpp biosynthesis and expression levels of the T3SS genes under nutritional stress.** (A) Serine hydroxamate (SHX)-induced (p)ppGpp biosynthesis. Wild type strain PA14 was grown in M9 medium supplemented with 0.2% casamino acids (CAA) to an OD_{600} of 1.0, and then incubated with 1 mM SHX for 45 min before imaging. (B) Wild type strain PA14 was grown in M9 medium supplemented with 0.2% CAA to an OD_{600} of 1, and then incubated with or without 1 mM SHX for 45 min. The mRNA levels of the T3SS gene were determined by real-time PCR. Data represent the mean ± standard deviation performed in triplicate and are representative of three independent experiments with similar results.

Supplemental Methods. Strain and plasmid construction.

Construction of the deletion mutant in *P. aeruginosa* was performed as described previously (1). To construct the *gltA* deletion mutant, a 1126 bp and a 1065 bp fragments upstream and downstream of the *gltA* coding region were amplified by PCR with primers *gltA*upF, *gltA*upR and *gltA*downF, *gltA*downR with PA14 chromosomal DNA as the template (Table S1). The PCR products were secondly amplified by PCR with primers *gltA*upF and *gltA*downR. The 2191 bp PCR products were cloned into the EcoRI-SmaI sites of the plasmid pEX18Tc, respectively. The resultant plasmid was transferred into the *E. coli* conjugation donor strain S17-1, and then transferred to PA14 through conjugation. The PA14 single-crossover mutants were selected on LB plates with 25 μg/mL kanamycin (to kill the S17-1 donor strain) and 100 μg/mL tetracycline. The single-crossover mutants were grown in LB for 16 hours, and then plated on LB plates containing 5% sucrose to select for double-crossover mutants. The correct deletion mutants were deleted 1287 bp fragments and a 2191 bp fragments were screened by PCR with primers *gltA*upF and *gltA*downR. For the deletion of *relA*, a 1071 bp and a 1022 bp fragments upstream and downstream of the *relA* coding region were amplified by PCR with primers *relA*upF, *relA*upR and *relA*downF, *relA*downR with PA14 chromosomal DNA as the template (Table S1). The PCR products were secondly amplified by PCR with primers *relA*upF and *relA*downR. The 2093 bp PCR products were cloned into BamHI- HindIII sites of the plasmid pEX18Tc, respectively. Construction of single- and double-crossover mutants as well as screening for *relA* deletion mutants were performed as

aforementioned. The correct deletion mutants were deleted 2244 bp fragments and a 2093 bp fragments were screened by PCR with primers *gltA*upF and *gltA*downR. Deletion of *spoT*, a 1020 bp and a 1011 bp fragments upstream and downstream of the *spoT* coding region were amplified by PCR with primers *spoT*upF, *spoT*upR and *spoT*downF, *spoT*downR with PA14 chromosomal DNA as the template (Table S1). The PCR products were secondly amplified by PCR with primers *spoT*upF and *spoT*downR. The 2031 bp PCR products were cloned into BamHI- HindIII sites of the plasmid pEX18Tc, respectively. Construction of single- and double-crossover mutants as well as screening for *spoT* deletion mutants were performed as aforementioned. The correct deletion mutants were deleted 2106 bp fragments and a 2031 bp fragments were screened by PCR with primers *spoT*upF and *spoT*downR.

For the complementation of *gltA* through chromosomal insertion, a fragment containing the promoter region and *gltA* open reading frame was amplified by PCR with primers *gltA*comF and *gltA*comR (Table S1). The 1749 bp PCR product was cloned into the BamHI-HindIII sites of the plasmid pUC18T-mini-Tn7T-Gm (2). The resultant plasmid was transferred into the Δ*gltA* mutant by conjugation. Selection of strains with the fragment insertion was performed as previously described (2).

To construct the P_{lacP1}-*gfp* transcriptional fusion, the *lac*P1 promoter (3) was amplified by PCR with primers *lac*P1F and *lac*P1R (Table S1) and cloned into the EcoRI- BamHI sites of the plasmid pDN19. The coding region of *gfp* was amplified by PCR with primers *egfp*F and *egfp*R (Table S1). The 717 bp PCR product was cloned into the BamHI-HindIII sites of the plasmid pDN19- P_{lacP1}.

To construct the P_{exsA}-*gfp* transcriptional fusion, the *exsA* promoter (4) was amplified by PCR with primers P_{exsA} F and P_{exsA} R (Table S1) and cloned into the EcoRI-BamHI sites of the plasmid pDN19. The coding region of *gfp* was amplified by PCR with primers *egfp*F and *egfp*R (Table S1). The 717 bp PCR product was cloned into the BamHI-HindIII sites of the plasmid pDN19- P_{exsA}.

References

1. Hoang TT, Karkhoff-Schweizer RR, Kutchma AJ, Schweizer HP. 1998. A broad-host-range Flp-FRT recombination system for site-specific excision of chromosomally-located DNA sequences: application for isolation of unmarked Pseudomonas aeruginosa mutants. Gene 212:77-86.

2. Choi KH, Schweizer HP. 2006. mini-Tn7 insertion in bacteria with single attTn7 sites: example Pseudomonas aeruginosa. Nat Protoc 1:153-61.

3. Fulcher NB, Holliday PM, Klem E, Cann MJ, Wolfgang MC. 2010. The Pseudomonas aeruginosa Chp chemosensory system regulates intracellular cAMP levels by modulating adenylate cyclase activity. Mol Microbiol 76:889-904.

4. Marsden AE, Intile PJ, Schulmeyer KH, Simmons-Patterson ER, Urbanowski ML, Wolfgang MC, Yahr TL. 2016. Vfr Directly Activates exsA Transcription To Regulate Expression of the Pseudomonas aeruginosa Type III Secretion System. J Bacteriol 198:1442-50.

Table S1. Bacterial susceptibilities to antibiotics

Strain	MIC（μg/ml)				
	Cip	TB	Azm	Mem	PB
PA14	0.25	2	150	0.25	0.625
Δ*gltA*	0.25	2	150	0.25	0.625
Δ*gltA*/att7::*gltA*	0.25	2	150	0.25	0.625

Cip, ciprofloxacin; TB, tobramycin; Azm, azithromycin; Mem, meropenem; PB, polymyxin B.

Table S2. Strains and plasmids used in this study.

Strain/ Plasmid	Description	Source (Reference)
P. aeruginosa		
PA14	Wild type strain of *P. aeruginosa*	(1)
Δ*gltA*	PA14 with *gltA* gene deleted	This study
Δ*gltA*/att7::*gltA*	Δ*gltA* with *gltA* inserted into the chromosome with mini-Tn7 insertion	This study
Δ*gltA*Δ*relA*	PA14 with both *gltA* and *relA* gene deleted	This study
Δ*relA*	PA14 with *gltA* gene deleted	This study
Δ*gltA*Δ*spoT*	PA14 with both *gltA* and *spoT* gene deleted	This study
**Plasmids**		
pUC18T-mini-Tn7T-Gm	mini-Tn7 base vector insertion into chromosome attTn7 site, Gm^r	(2)
pDN19	Shuttle vector between *E. coli* and *P. aeruginosa*; Tc^r	(3)
pEX18Tc	Gene knockout vector; Tc^r	(2)
pUCP20 (promoterless)	Shuttle vector between *E. coli* and *P. aeruginosa*; Amp^r	Laboratory stock
**Primer**	**Sequence（5'→3'）**	**Function**
*gltA*upF	CCGGAATTCCAAAGGAAAGTGTACGAATGCT	*gltA* deletion
*gltA*upR	GCCTTTCTCGCCAGGTGGCCTCCTATTTATGCTTGA	*gltA* deletion
*gltA*downF	ATAAATAGGAGGCCACCTGGCGAGAAAGGCTGC	*gltA* deletion
*gltA*downR	TCCCCCGGGGCTGCATAGCACCGTCGATT	*gltA* deletion
*gltA*comF	CGGGATCC GAACAGGATGACGCCAGAG	*gltA* complementation

*gltA*comR	CCAAGCTT TCAGCCGCGATCCTTG	*gltA* complementation
*relA*upF	CGGGATCC CAGCGTTTCGCCGGGA	*relA* deletion
*relA*upR	TGTTTCCGCCTCGCCCTTGCCTACCCTTTACCACGG	*relA* deletion
*relA*downF	GTAAAGGGTAGGCAAGGGCGAGGCGGAAACAGG	*relA* deletion
*relA*downR	CCAAGCTT GCGGTGATCCTGCTGGTC	*relA* deletion
*spoT*upF	CGGGATCCCTGTACATCGTTTCCGCTCCC	*spoT* deletion
*spoT*upR	CAGGAAAAAGCGGGGGGTTCACCCCCTGCCC	*spoT* deletion
*spoT*downF	CAGGGGGTGAACCCCCCGCTTTTTCCTGTGTCAT	*spoT* deletion
*spoT*downF	CCAAGCTTGGCAGACCGCTGACCAG	*spoT* deletion
*lac*P1F	GAATTCGCCCAATACGCAAACCGC	*lac*P1 promoter cloning
*lac*P1R	GGATCCTCAGGCGAAAGGGGGATGTGCTG	*lac*P1 promoter cloning
P_{exsA} F	GAATTCGCATTGTACCTACGCACCG	*exsA* promoter cloning
P_{exsA} R	GGATCCTTATCTGCTTTCGGCCAAG	*exsA* promoter cloning
*egfp*F	CGGGATCCAAAGAGGAGAAATTAACCATGGGTA	*egfp* cloning
*egfp*R	CCAAGCTTTTATTTGTATAGTTCATCCATGCCA	*egfp* cloning

q*exsA*F	GGTAAACAAGGAAGAGGGCGTA	RT-PCR
q*exsA*R	GGACGAAGCCTTGTAGAAACTG	RT-PCR
q*exsC*F	ATCGGTTTGCCTTCCCTGTC	RT-PCR
q*exsC*R	AAAGATCCCCTCGCCCAG	RT-PCR
q*exsD*F	AGAGGTGCGGCAGATTCTCC	RT-PCR
q*exsD*R	GCAGCAGGACCCAATCGA	RT-PCR
q*pcrV*F	CCCACGCTCTATGGCTATGC	RT-PCR
q*pcrV*R	TTGAGTTCCCCGCTCTGCT	RT-PCR
q*sodB*F	CTTCTACTGGAACTGCCTGAGC	RT-PCR
q*sodB*R	GAACTTGTCGAAGGAGCCGA	RT-PCR
q*rpoS*F	GGACTCGGACAAGACCCTG	RT-PCR
q*rpoS*R	CACCTCACGCTGCTTGTCG	RT-PCR
qPA1805F	ATTTCGATCCATCCATGCTG	RT-PCR
qPA1805R	AAGCGATCCGCGTTGAAC	RT-PCR

Gm^r, gentamycin resistance; Amp^r, ampicillin resistance; Tc^r, tetracycline resistance;

References

1. Liberati NT, Urbach JM, Miyata S, Lee DG, Drenkard E, Wu G, Villanueva J, Wei T, Ausubel FM. 2006. An ordered, nonredundant library of Pseudomonas

aeruginosa strain PA14 transposon insertion mutants. Proc Natl Acad Sci U S A 103:2833-8.

2. Choi KH, Schweizer HP. 2006. mini-Tn7 insertion in bacteria with single attTn7 sites: example Pseudomonas aeruginosa. Nat Protoc 1:153-61.

3. Li K, Xu C, Jin Y, Sun Z, Liu C, Shi J, Chen G, Chen R, Jin S, Wu W. 2013. SuhB is a regulator of multiple virulence genes and essential for pathogenesis of Pseudomonas aeruginosa. mBio 4:e00419-13.
